# Infectious endocarditis during pregnancy, problems in the decision-making process: a case report

**DOI:** 10.4076/1757-1626-2-6537

**Published:** 2009-09-10

**Authors:** Enrico Vizzardi, Giuseppe De Cicco, Gregoriana Zanini, Antonio D’Aloia, Pompilio Faggiano, Roberto Lo Russo, Ermanna Chiari, Livio Dei Cas

**Affiliations:** 1 Department of Applied Experimental Medicine, Section of Cardiovascular Disease, Brescia Study UniversityP.le Spedali Civili, 1-BresciaItaly; 2 Department of Cardiac Surgery, Spedali Civili BresciaP.le Spedali Civili, 1Italy

## Abstract

Infective endocarditis in pregnancy has a low incidence, often being associated with a previous history of rheumatic or congenital heart disease. In most reports the disease tends to run a subacute course and to appear more frequently in the third trimester of pregnancy. We present the case of a 36-year-old woman with large vegetations on the mitral valve due to infective endocarditis detected at the 32^nd^ week of her first pregnancy. The difficulties in selecting the appropriate management strategy, particularly optimal time and mode of delivery, optimal time and type of valve surgery, are emphasized.

## Introduction

The incidence of infective endocarditis during pregnancy is reported as low (0.006%) [1,2]. Maternal mortality rate, however, is significant high (33%), most deaths being related to heart failure or an embolic event [3]; even the rate of foetal mortality can reach 29%. Infective endocarditis in pregnancy can present difficulties in management, such as the choice of appropriate antibiotic treatment and the choice of optimal time for delivery and cardiac surgery [4]. The case report we present here clearly illustrates these therapeutic dilemmas.

## Case presentation

A 36-year-old Caucasian woman at the 32^nd^ week of gestation presented with a few days history of asthenia and pharyngodynia without fever. Her previous medical history reported the presence of mitral valve prolapse. Vital signs at admission included normal body temperature (36.7°C), a pulse rate of 110 beats/min, a blood pressure of 130/70 mm Hg and a respiratory rate of 24 breaths/min; physical examination detected a 2/6 systolic murmur.

The electrocardiogram showed sinus tachycardia without specific ST and T-wave changes. Laboratory tests revealed mild anaemia (haemoglobin 11.1 g/dl) and leukocytosis with neutrophilia (white blood cell count 22,600/mm^3^). C-reactive protein (CRP) was markedly elevated (89.7 mg/dl), and first-hour erythrocyte sedimentation rate (ESR) was 110 mm.

**Figure 1.**
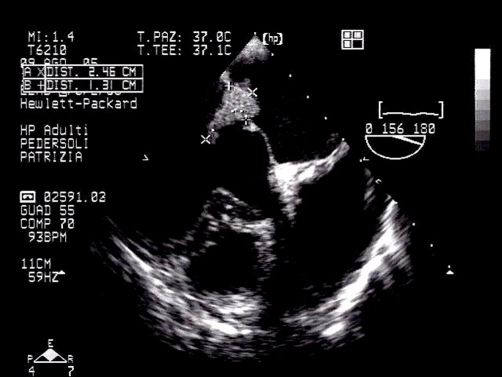
Transoesophageal echocardiography with the evidence of large vegetation attached to the atrial side of the anterior mitral leaflet.

Transthoracic echocardiography showed a huge mass on the mitral valve, and a transoesophageal echocardiogram was performed immediately (Figure 1) to clarify this finding. This revealed a large vegetation attached to the atrial side of the anterior mitral leaflet, with a long mobile component occupying the valve orifice and the left ventricular inflow tract during diastole; the posterior leaflet was thickened and retracted. As a consequence of posterior leaflet abnormalities, anterior leaflet prolapse and the attached mass, moderate to severe regurgitation and mild stenosis were detected at colour Doppler examination.

Ultrasound examination showed an appropriate for gestational age foetus in vertex presentation. In view of the patient’s clinical condition, IV antibiotic therapy was not started before the results of the blood cultures were known. Blood cultures were positive for Streptococcus Species so antibiotic therapy (ampicillin + sulbactam 12 g) was commenced.

The obstetrician was of the opinion that the pregnancy should be terminated but was not sure whether a caesarean section was the best option; the cardiac surgeon consulted for the therapeutic approach was more in favour of a few days’ antibiotic treatment prior to surgery, despite being aware that it was a case at high risk of embolism.

The third day after admission, however, the patient’s membrane ruptured spontaneously and she delivered a female infant after a gestation of 33 weeks and 3 days.

Two days after delivery, the patient complained of left leg pain; an ultrasound examination revealed thrombosis of the popliteal artery so she underwent successful percutaneus embolectomy. Based on this serious event and in order to prevent further embolism, an immediate surgical approach was planned. On the same day, she underwent to the surgical removal of the vegetation on the mitral valve and valve commissurotomy. A histological examination of specimens of the resected leaflets indicated that infective endocarditis was active. The intraoperative Doppler examination revealed trivial mitral regurgitation and a mitral valve area of 2.5 cm^2^. Following surgical repair of the mitral valve, antibiotic treatment was switched from ampicillin-sulbactam to ciprofloxacin and ampicillin for 4 weeks.

At six month follow-up visit both the patient and the newborn were in good clinical conditions.

## Discussion

The presence of valvular heart disease during pregnancy poses a challenge to both physician and patient, increasing the risk of unfavourable maternal and/or foetal outcomes. Nazarian et al [2] first described a successful outcome in the case of a pregnant woman with bacterial endocarditis in 1976. Bacterial endocarditis in pregnancy is extremely rare, and is either a complication of a pre-existing cardiac lesion or the result of intravenous drug use. Cox et al [3,5,6] describe their experience with seven women whose pregnancies were complicated by endocarditis. Streptococcus viridans was the most commonly isolated organism (found in four out of seven cases). In a recent study, the maternal and foetal mortality rate calculated for pregnancy-associated endocarditis was 22% and 15%, respectively [7]. Active endocarditis is a serious, life-threatening condition, and surgical treatment is usually delayed until the infection has been eliminated. Valve replacement in the presence of active infection leads to a high risk of re-infection. Emergency surgical treatment must be performed if shock, severe heart failure or persistent embolic events are present. Our case report describes an extremely rare condition, in which a pregnant women developed bacterial endocarditis of a mitral valve prolapse from Streptococcus species infection and underwent successful valve repair. Close attention should be paid to any pregnant woman with an unexplained fever and a cardiac murmur or pre-existent heart disease. Rapid detection of endocarditis and appropriate treatment are important in reducing the risk of both maternal and foetal mortality.

In the clinical management of pregnant women with endocarditis, the most important issue is to save the lives of both mother and child. Heart surgery is not recommended during the first two trimesters, except in extreme emergencies [8]. Furthermore, recent advances in neonatal care have improved the survival of premature infants born after at least 28 weeks of gestation. For these reasons, elective delivery by caesarean section just prior to cardiopulmonary bypass has been advocated to minimize maternal and foetal risks [8]. There are, however, no published studies in support of caesarean section or natural childbirth in women suffering from endocarditis, and even European guidelines provide no useful information on the matter [4].

The problem is the choice of correct management: birth induction followed by antibiotic therapy and cardiac surgery, or specific antibiotic therapy followed by gestation with concomitant cardiac surgery. We believe that the decision to perform early delivery by caesarean section and intervene on the patient’s heart at a later stage is the most logical strategy when emergent open heart surgery is inevitable for the treatment of cardiac disease and in cases of at least 24 weeks’ gestation. Based on published data [9-10], we maintain that valve repair is not only feasible but also safer than valve replacement in the early stage of endocarditis, but it is particularly important to remember that in cases such as the one described here the fundamental thing is making a swift decision.

**Figure 2.**
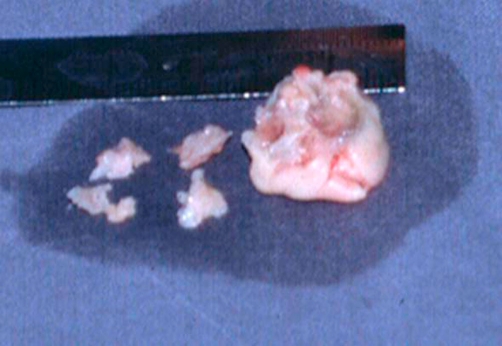
Anatomical view of the specimens of the resected leaflets.

## Abbreviations

CRP: C-reactive protein
ESR: first-hour erythrocyte sedimentation rate
